# IκB-kinase-ε in the tumor microenvironment is essential for the progression of gastric cancer

**DOI:** 10.18632/oncotarget.20778

**Published:** 2017-09-08

**Authors:** Biao Geng, Chen Zhang, Chao Wang, Ying Che, Xianmin Mu, Jinshun Pan, Che Xu, Shi Hu, Jing Yang, Ting Zhao, Yue Xu, Yuanfang Lv, Hao Wen, Zheng Liu, Qiang You

**Affiliations:** ^1^ Department of Biotherapy, Second Affiliated Hospital, Nanjing Medical University, Nanjing, Jiangsu 210011, China; ^2^ Department of Surgery, Second Affiliated Hospital, Nanjing Medical University, Nanjing, Jiangsu 210011, China; ^3^ Medical Center for Digestive Diseases, Second Affiliated Hospital, Nanjing Medical University, Nanjing, Jiangsu 210011, China; ^4^ Key Laboratory for Aging & Disease, Second Affiliated Hospital, Nanjing Medical University, Nanjing, Jiangsu 210011, China; ^5^ Department of Immunology, Nanjing Medical University, Nanjing, Jiangsu 211166, China

**Keywords:** gastric cancer, IκB-kinase-ε, tumor-infiltrating lymphocytes, metastasis, prognostic biomarker

## Abstract

The tumor microenvironment is critical for tumor growth and metastasis, but the underlying molecular mechanisms are poorly understood. Recent studies have shown that IκB-kinase-ε (IKKε) is involved in the proliferation and migration of certain cancers. However, the functional role of IKKε in the progression of gastric cancer (GC) remains unknown. In this study, we found that high levels of IKKε expression in GC tumors were correlated with more advanced disease and poor overall survival of patients. Silencing of IKKε effectively suppressed the migratory and invasive capabilities of human GC cells *in vitro* and tumorigenicity and metastasis *in vivo*. Further analysis revealed that IKKε was also highly expressed in tumor-infiltrating lymphocytes. Moreover, it was involved in tumor-infiltrating T-cell-mediated invasion and metastasis. Knockdown of IKKε elevated T-cell antitumor immunity. These findings suggest that IKKε may be a novel prognostic marker and a potential therapeutic target in human GCs.

## INTRODUCTION

Gastric cancer (GC) is the leading cause of cancer-related death in China [1]. At the time of initial diagnosis, a large majority of patients have already reached an advanced stage in which tumor cell spreading has occurred, and approximately 50% of patients with GC will die from the development of distant metastases [2, 3]. To improve the early diagnosis of GC and targeted therapy, an in-depth understanding of the molecular underpinnings of the disease is required [4]. It is of clinical importance to identify genes that contribute to GC development and present predictive values for diagnosis or prognosis.

The nuclear factor (NF)-κB pathway is a pivotal regulator of several important physiological functions, including the inflammatory immune response, proliferation, cell survival and cell invasion [5, 6]. These activities are well-described hallmarks of cancer, and NF-κB activation has been observed in a wide range of tumors, leading some to suggest that NF-κB serves as a bridge between inflammation and cancer [7-10]. I-κB kinases (IKKs), IKKε and TBK1, are key regulators of NF-κB signaling [5]. The IKK-related kinases have recently been recognized as NF-κB effectors that contribute to tumorigenesis and thus represent a link between NF-κB-mediated inflammation and cancer [11-13]. Studies in animal models have shown that NF-κB is often essential for cancer initiation and progression [11, 14]. Previous studies have suggested that IKKε is overexpressed in tumor tissue from a variety of human tumor types, such as breast cancer, non-small cell lung cancer, and pancreatic cancer, where it has been proposed as biomarker and potential therapeutic target [15-18]. However, a comprehensive understanding of how IKKε promotes tumorigenicity is lacking. IKKε is also expressed in immune cells, and may play a special role in the immune response [11, 19, 20]. Furthermore, IKKε is a crucial negative regulator of T-cell activation and a potential target for immunotherapy [21]. However, the function of IKKε remains obscure in tumor-infiltrating regulatory T-cells, despite its abundant expression. Therefore, we set out to more fully understand how IKKε controls tumor-infiltrating lymphocyte crosstalk in GC metastasis.

In the present study, we report that, in addition to the fact that IKKε is aberrantly overexpressed in GC and could coordinately serve as a promising predictive biomarker for prognosis in patients with GC, knockdown of IKKε elevates T-cell antitumor immunity and reduces tumor development. Furthermore, the functional role of IKKε in GC provides a mechanistic basis for its potential as a therapeutic target.

## RESULTS

### IKKε is upregulated in GC tissues and is correlated with GC progression

**Figure 1:**
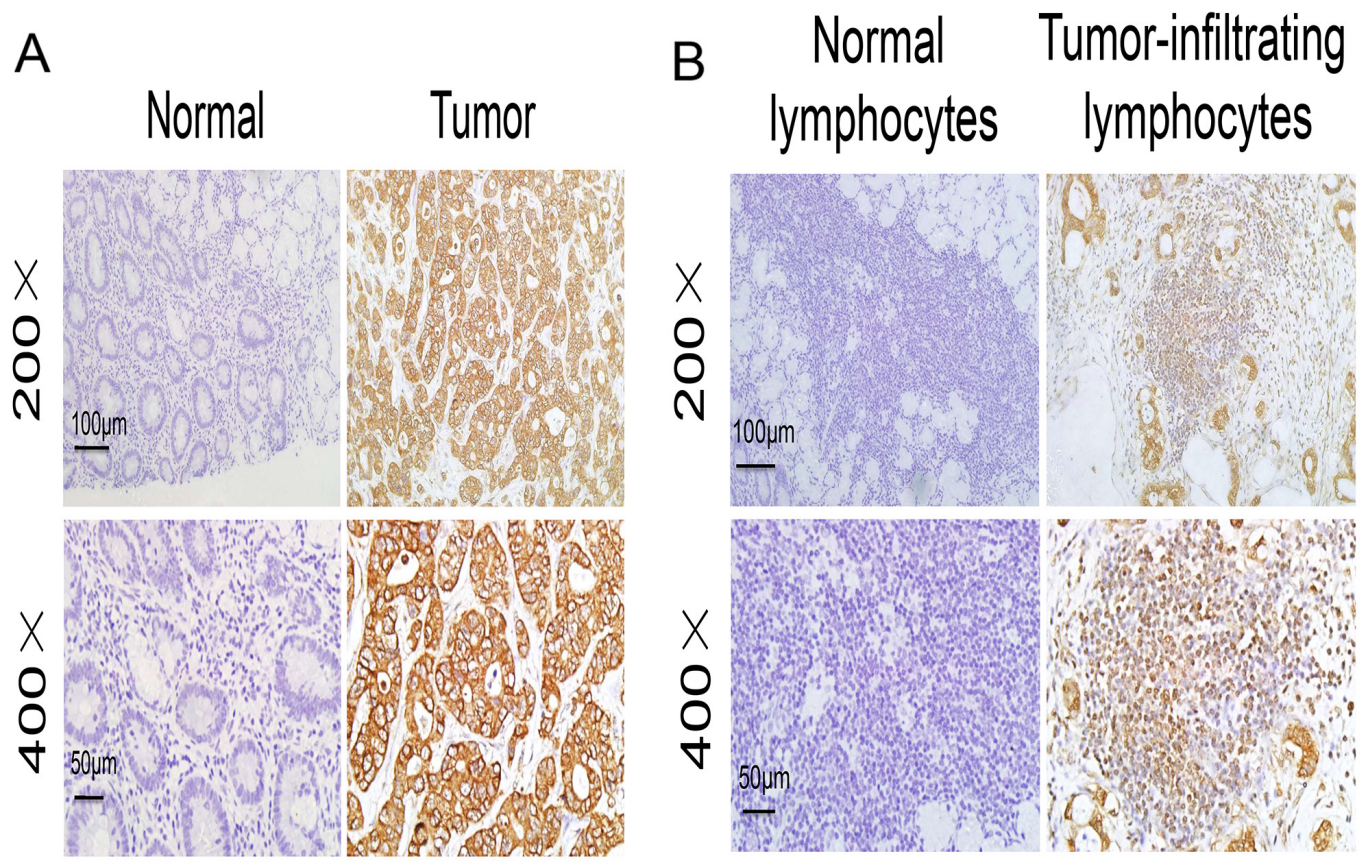
IKKε expression in the tumor microenvironment **(A)** Representative images of IKKε staining in adjacent non-tumor tissues and GC tissues. **(B)** Representative images of IKKε staining in lymphocytes from adjacent non-tumor tissues and tumor-infiltrating lymphocytes from GC tissues.

To examine the significance of IKKε in GC development, we first measured IKKε expression in 2 GC samples using IHC. IKKε was significantly upregulated in GC tissues compared with adjacent non-cancerous gastric tissues (Figure 1A). Furthermore, compared with lymphocytes from adjacent non-tumor tissues, there was an obvious increase of IKKε expression in tumor-infiltrating lymphocytes from GC tissues (Figure 1B). To further investigate the association of IKKε and GC progression, tissue microarray-based IHC study of IKKε in 100 GC tissues with clinicopathological features and complete follow-up data was performed. As shown in Table 1, high expression of IKKε was found to be significantly associated with poor differentiation (*P* = 0.021), depth of invasion (*P* = 0.034), lymph node metastasis (*P*< 0.001), distant metastasis (*P* = 0.006), and tumor-node-metastasis (TNM) stage (*P* = 0.005).

**Table 1:** Association between IKKε expression and clinicopathological factors in gastric cancer

Variable	n	IKKε expression		*P* value
		Low	High	
**Age**				0.610
≤ 60	43	18	25	
> 60	57	21	36	
**Gender**				0.674
Male	72	29	43	
Female	28	10	18	
**Differentiation**				0.021
Well	12	9	3	
Moderate	65	21	44	
Poor	23	9	14	
**Depth of invasion (T)**				0.034
T1-T2	36	19	17	
T3-T4	64	20	44	
**Lymph node metastasis**				0.000
N0-N1	33	28	15	
N2-N3	67	11	46	
**Distant metastasis (M)**				0.006
Negative (M0)	78	36	42	
Positive (M1)	22	3	19	
**Tumor stage**				0.005
I-II	37	21	16	
III-IV	63	18	45	

**Figure 2:**
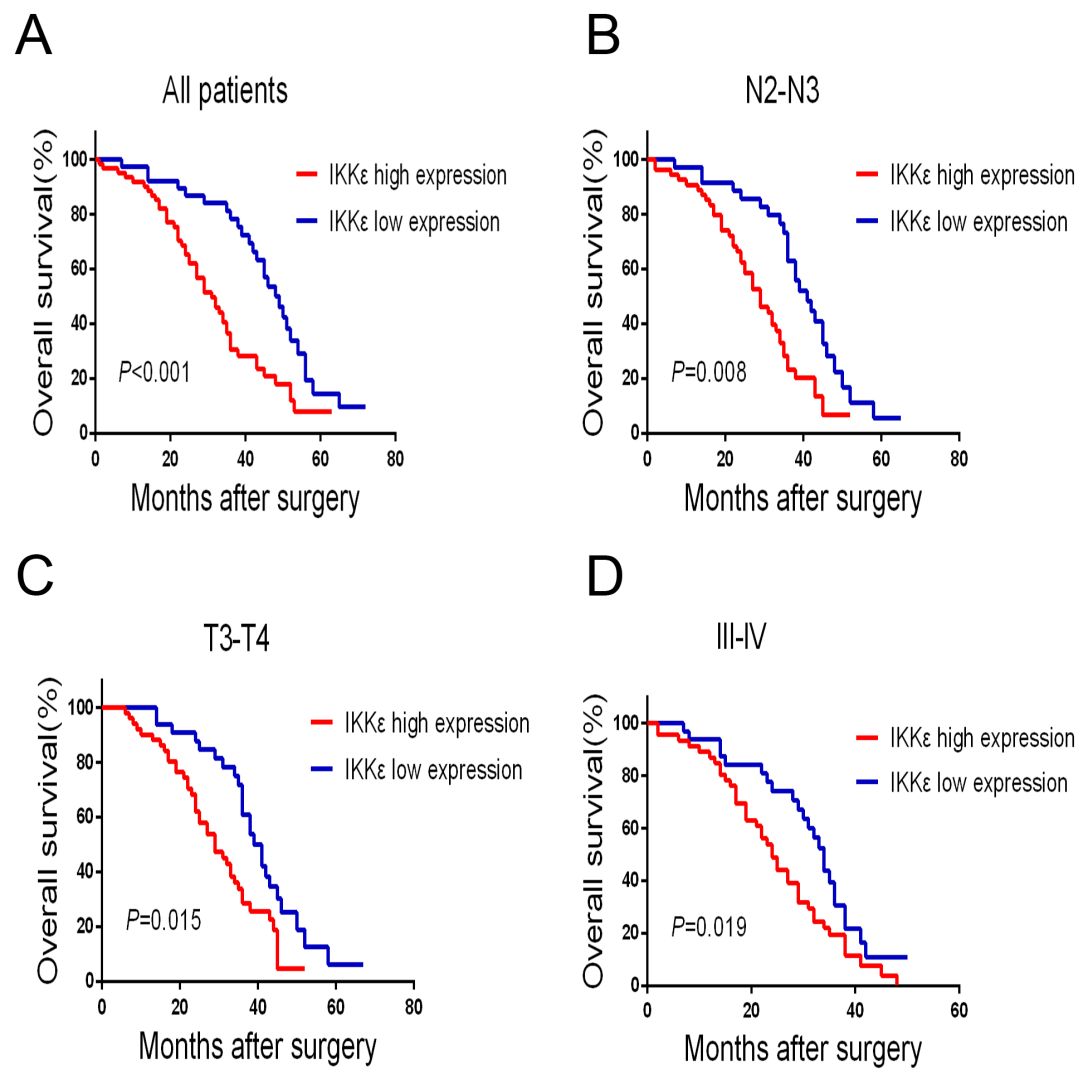
IKKε overexpression is associated with poor prognosis in GC patients **(A)** Kaplan–Meier analysis for OS of patients with gastric cancer according to IKKε expression in all patients, patients with N2-N3 stage tumors **(B)** patients with T3-T4 stage tumors **(C)**, and patients with III-IV stage tumors **(D)**.

To estimate the clinical prognostic significance of IKKε expression that might influence the overall survival of patients enrolled in this study, Kaplan-Meier survival analysis was performed in the cohort. As shown in Figure 2, patients with higher expression of IKKε in tumor tissues were prone to lower overall survival (OS). Low expression of IKKε has a survival benefit compared with high expression (Figure 2A, *P*<0.001). Kaplan-Meier analysis was also applied to compare overall survival according to IKKε expression in different clinicopathological factors. Significant differences were found in N2-N3, T3-T4, and III-IV stage tumors according to IKKε expression (Figure 2B, *P* = 0.008, Figure 2C, *P* = 0.015, Figure 2D, *P* = 0.019). Moreover, Cox regression analysis also indicated that high IKKε expression was an independent prognostic factor for poor survival in GC patients (Table 2). Together, these results suggest that IKKε overexpression was significantly associated with poor prognosis of GCs.

**Table 2:** Multivariate analysis for overall survival

Variable	HR	CI (95%)	P value
Depth of invasion	1.236	0.924–2.256	0.121
Lymph node metastasis	2.374	1.237–3.562	0.026
Distant metastasis	4.518	2.331–8.318	0.001
Tumor stage	5.231	3.169–7.573	0.000
Differentiation	1.252	0.726–1.842	0.535
IKKε expression	2.013	1.204–7.471	0.014

### IKKε regulates cell proliferation, migration, and invasion

**Figure 3:**
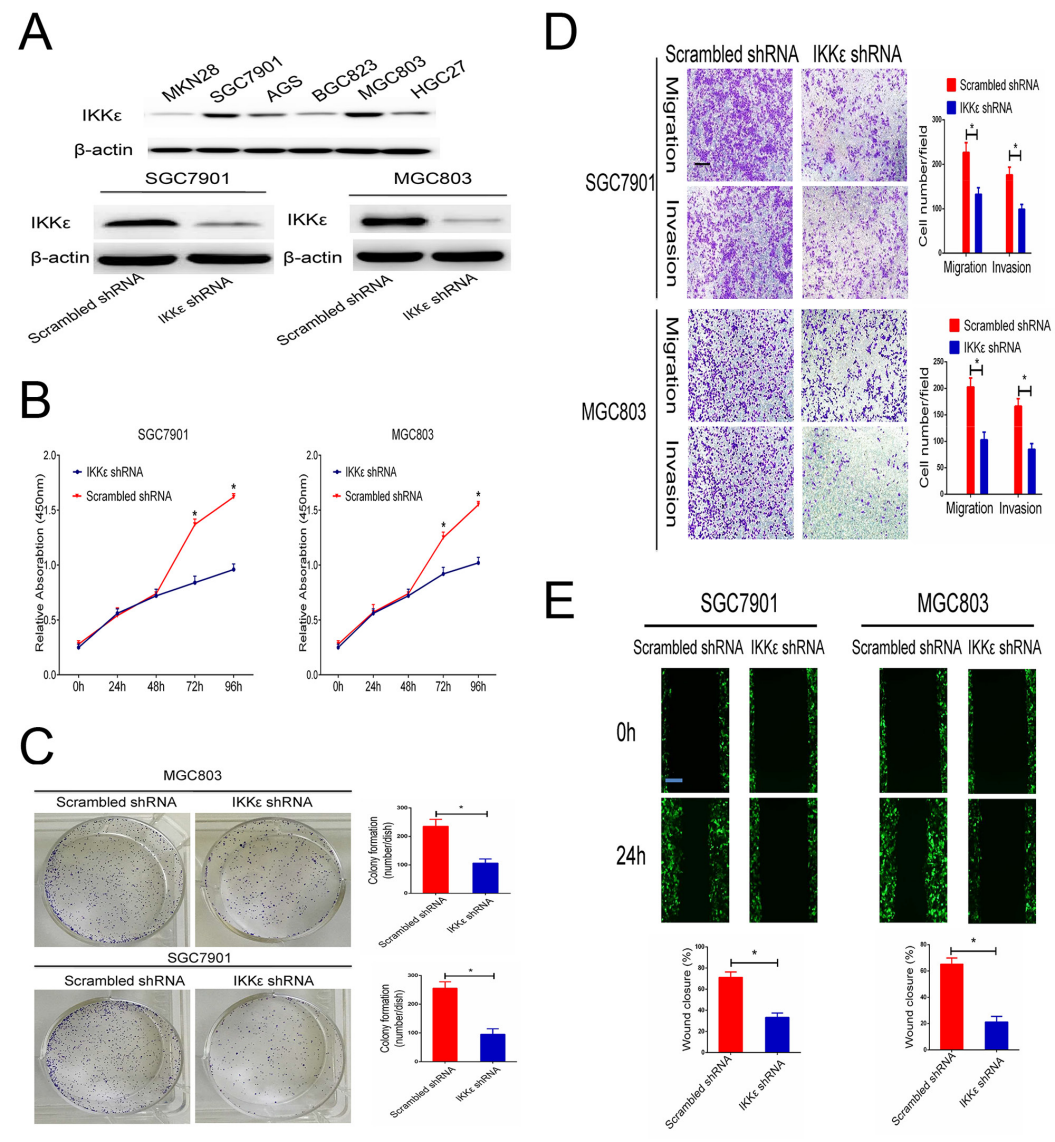
IKKε enhances the invasion and migration of GC cell lines **(A)** The expression of IKKε was analyzed by Western blotting GC cell lines. SGC7901 and MGC803 cells were transfected with IKKε shRNA or scrambled shRNA. The efficacy of knockdown was assessed by Western blot. **(B)** CCK-8 assays were used to analyze the proliferation of GC cells transfected with IKKε shRNA or scrambled shRNA. **(C)** Plate clone formation efficiencies of cells in the presence of IKKε shRNA were compared with negative controls. **(D)** Transwell assay in SGC7901 and MGC803 cells stably transfected with IKKε shRNA or scrambled shRNA. Scale bars, 100μm. **(E)** Wound healing assays for SGC7901 and MGC803 cells transfected with IKKε shRNA or scrambled shRNA. Scale bars, 100μm. Data from 3 independent experiments were presented as mean ± SD. *P<0.05.

We first examined the expression level of IKKε in a panel of human GC cells. The results indicated that the protein expression of IKKε was higher in SGC7901and MGC803 cells (Figure 3A). To identify the potential function of IKKε in GC cell growth and metastasis, SGC7901 and MGC-803 cells with stably knocked-down IKKε were created. Changes in IKKε expression were confirmed using western blotting (Figure 3A). The analysis of cell proliferation by the CCK8 assay revealed that compared with control cells, IKKε knockdown cells had lower proliferation (Figure 3B) and colony-formation rates (Figure 3C). The number of cells that invaded through the Matrigel or migrated was clearly decreased for IKKε knockdown cells in the Transwell assay compared to control cells (Figure 3D). The wound closure assay verified the results, which showed that wound recovery was significantly impaired by IKKε knockdown in comparison with the controls (Figure 3E). Together, these experiments identified IKKε as the critical oncoprotein that mediates GC cell proliferation and invasion.

### Knockdown of IKKε elevates T cell antitumor immunity and reduces tumor development

**Figure 4:**
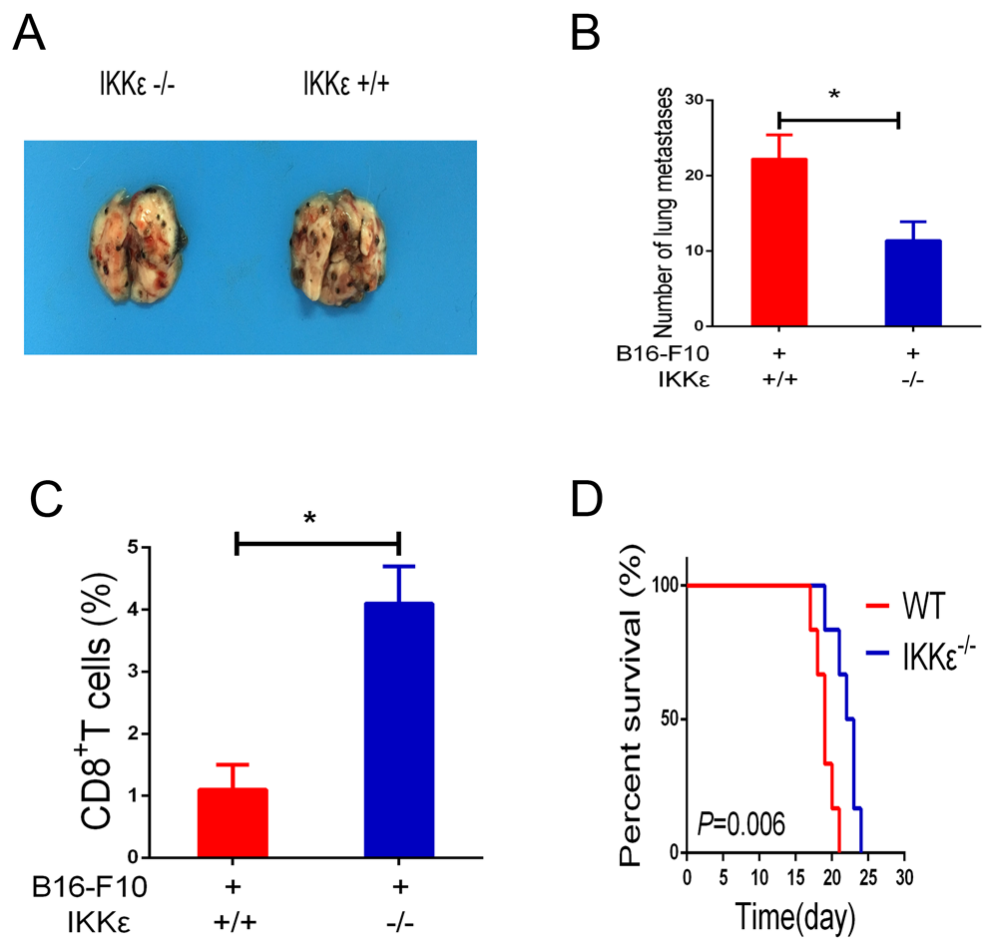
Knockdown of IKKε elevates T-cell antitumor immunity **(A)** WT mice and IKKε null (-/-) mice received B16-F10 melanoma cells by tail-vein injection. Two weeks later, melanoma metastasis was visually assessed (n=6/group). **(B)** Quantification of pleural melanoma colonies. **(C)** The isolated cells from bronchoalveolar lavage fluid (BALF) were analyzed by CD8 staining. **(D)** Mouse survival was shown by Kaplan–Meier survival curves. *P<0.05.

Recent studies have demonstrated that IKKε is expressed in immune cells and may play a special role in regulating T-cell function [11, 14, 21]. As a consequence, studies were undertaken to determine whether IKKε upregulation plays a role in promoting tumor metastasis. To accomplish this, we compared the B16-F10 melanoma cell-induced metastasis in WT mice and IKKε null mice. Melanoma cell administration caused impressive levels of metastasis in the lungs of WT mice, and this metastatic response was markedly decreased in IKKε null mice (Figure 4A, 4B). To investigate whether knockdown of IKKε influences T-cell function, we examined the content of CD8^+^ T-cells in bronchoalveolar lavage fluid. The results revealed that CD8^+^ T-cells in the WT mice were significantly decreased compared to mice with null mutations of IKKε (Figure 4C). Additionally, the survival of the IKKε null mice was increased compared with WT controls (Figure 4D). When viewed together, these studies demonstrate that knockdown of IKKε results in elevated numbers of activated CD8^+^ T-cells in the tumor microenvironment.

### IKKε promotes tumor growth, invasion, and metastasis *in vivo*

**Figure 5:**
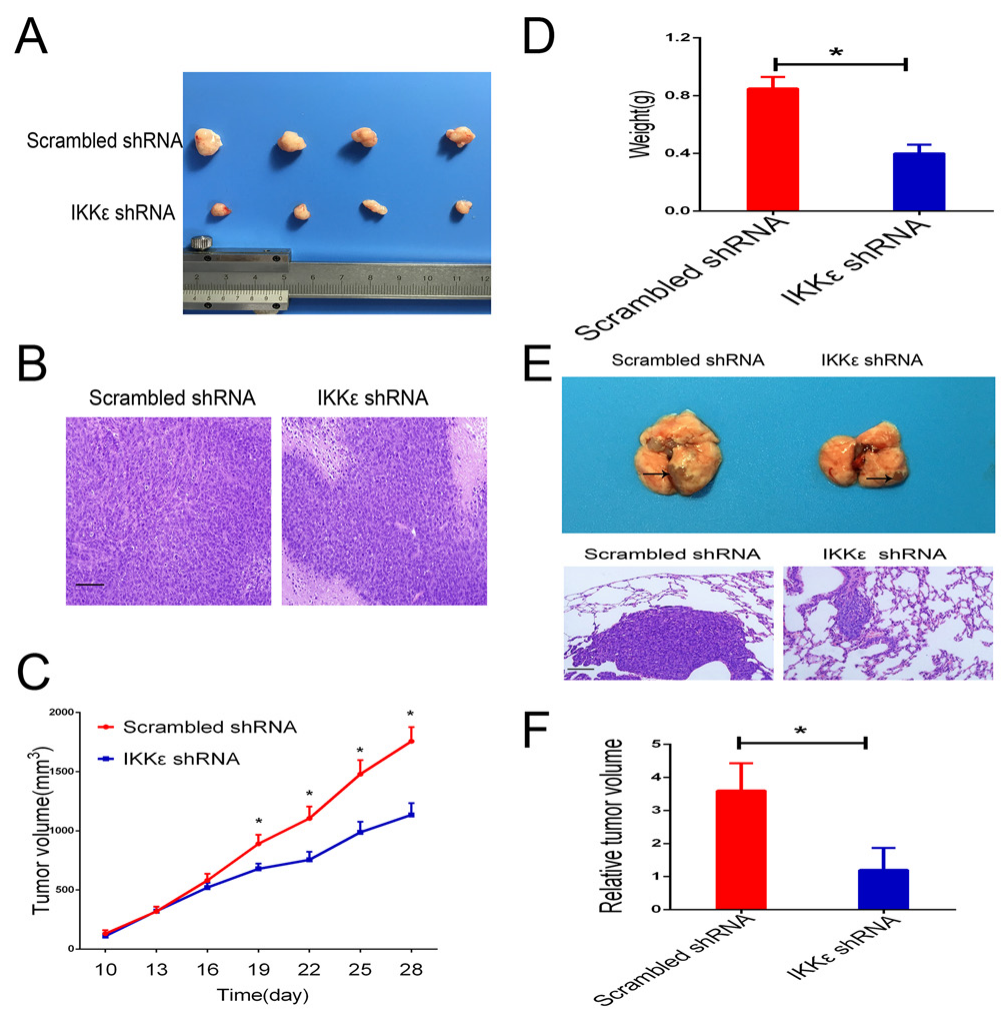
IKKε drives tumor growth and metastasis *in vivo* **(A)** Representative images of tumors formed in nude mice injected subcutaneously with MGC803 cells transfected with IKKε shRNA or control shRNA (n=8/group). **(B)** Representative images of tumor samples stained with hematoxylin and eosin (H&E) (200 × magnification). Scale bars, 100μm. **(C)** Quantification of tumor growth curves of xenograft in mice. **(D)** Quantification of tumor weights of xenograft in mice. **(E)** Representative images of metastatic tumors in lung samples (n=5/group). H&E staining of the representative metastatic lesions in nude mice (200 × magnification). Scale bars, 100μm. **(F)** The lungs were divided into 8 parts, and 5 slides acquired from each part were used to calculate the area of metastatic lesions. The total area of invasive lesions on these slides was described as the invasive tumor volume. *P <0.05.

To confirm these results of experiments *in vitro*, we further evaluated the functional role of IKKε expression on *in vivo* tumor growth and metastasis of GC cells. We first developed subcutaneous xenograft tumor models in nude mice by subcutaneous injection of MGC803 cells infected with scrambled or IKKε shRNA. As shown in Figure 5A and 5C, the size of the xenograft tumors derived from the IKKε knockdown MGC803 cells was significantly smaller than those formed by control cells. The weights of the xenograft tumors corresponded to their sizes (Figure 5D).

To investigate whether IKKε promoted tumor metastasis *in vivo*, we injected SGC7901 cells into the lateral tail vein of nude mice for assessment of metastasis. Histological examination validated pulmonary metastasis (Figure 5E). We found that IKKε knockdown significantly reduced the area of metastatic lesions that appeared in the lungs of the mice (Figure 5F).

Considering these data together, our experiments revealed that reduction of IKKε protein expression effectively interfered with the potential of GC cells to proliferate and metastasize *in vivo*.

## DISCUSSION

GC metastasis is the main cause of GC-related mortality. However, its mechanism remains poorly understood. It is a complex multistep process, involving alterations in the dissemination, invasion, survival, and growth of new cancer cell colonies, which are regulated by a complex network of intra- and inter-cellular signal transduction cascades [23, 24]. In this study, we have disclosed that IKKε upregulation is significantly associated with a more aggressive tumor phenotype. Cox regression analysis also indicated that high IKKε expression is an independent prognostic factor for poor survival in GC patients. We also revealed the underlying mechanism for such an association by demonstrating IKKε as a crucial negative regulator of T-cell activation.

IKKε’s play essential roles as regulators of proper immune function, cell survival, apoptosis, and cellular proliferation by modulating the NF-κB pathway [25]. It is critical for activation of NF-kB complexes downstream of cytokine signaling and through oncoprotein expression [26]. Recent studies have also implicated IKKε’s impact on cell proliferation and transformation, and it is thereby also classified as an oncogene [5]. It has been suggested that IKKε is aberrantly expressed in approximately 30% of breast carcinomas, in which it induces survival signaling associated with NF-κB pathway activation [18]. Furthermore, IKKε-associated cytokine signaling promotes tumorigenicity of immune-driven triple-negative breast cancers (TNBCs) [15]. Indeed, IKKε’s have emerged as a critical modulator of cancerous traits, yet little is known about its significance and expression in GC. Here, we have demonstrated that high expression of IKKε was found to be significantly associated with GC progression. Therefore, IKKε has potential for utilization as a predictive marker for GC patient outcomes. The tumor-facilitating functions of IKKε that have been described so far include the following: IKKε may contribute to enhanced NF-kB activity and tumorigenesis by directly phosphorylating NF-kB p65 or by phosphorylating Akt, which then phosphorylates and activates p65 [27]. Moreover, elevated IKKε directly phosphorylates and activates specific STAT transcription factors in different primary tumors and cell lines derived from a diversity of cancers, such as lung and breast carcinoma, which may contribute to the oncogenic activation of IKKε [17, 18, 27].

To further understand the biological function of IKKε in GC progression, we investigated the malignant features of IKKε in GC cell lines. Our data showed that knockdown of IKKε completely reversed the effect of IKKε in proliferation, colony formation, and migration and invasion assays. Decreased IKKε expression also reduced the growth and metastasis of GC xenografts in nude mice. These *in vivo* findings correlate well with *in vitro* results that IKKε functions as an inducer of GC metastasis and is correlated with clinical stage, lymph node metastasis, and prognosis in GC patients.

The tumor microenvironment, consisting of extracellular matrix (ECM), fibroblasts, vasculature, and tumor-infiltrating lymphocytes, is critical for tumor growth and metastasis [28-30]. Recently, an increasing amount of research has focused on the tumor microenvironment as it has become apparent that an environment rich in immune system cells, such as macrophages, T-cells, and B cells, promote tumor growth, metastasis, and relapse [24, 31-33]. In this study, we observed that that loss of IKKε results in elevated numbers of activated CD8^+^ T-cells in the tumor microenvironment. Conceivably, depletion of IKKε may elevate these signaling events to promote CD8^+^ T-cell activation. Our data showed that most patients with lymph node metastasis were in the IKKε-high group, whereas patients without lymph node involvement were in the IKKε-low group. One possible explanation for these observations is that cancer cells might secrete a paracrine factor or factors that promote IKKε expression in tumor-infiltrating T-cells, which would inhibit T-cell immune response. Thus, the mechanism of IKKε activation, in particular, and its roles in tumor-infiltrating lymphocytes in T-cells, in general, requires investigation.

These results identify 2 major clinically relevant directions for future work. First, IKKε overexpression is significantly correlated with more advanced disease and poor survival of GC patients. Thus, IKKε could serve as a promising predictive biomarker for recurrence and prognosis in patients with GC. Second, loss of IKKε elevates T-cell antitumor immunity, which may provide a path to combined therapy that would be effective in primary tumors or established metastasis.

## MATERIALS AND METHODS

### Cell culture

Human GC cell lines MKN28, AGS, SGC7901, BGC823, MGC803, and HGC27 (Cell Bank of Shanghai Institute of Cell Biology, Chinese Academy of Sciences) were cultured in RPMI 1640 medium (Sigma, USA) supplemented with 10%fetal bovine serum (FBS) (Gibco, USA), 100 units/ml penicillin, and 100 mg/ml streptomycin (Thermo Scientific). Cells were cultured at 37°C in a humidified 95% air, 5% CO_2_atmosphere.

### Immunohistochemistry (IHC)

A GC tissue microarray containing 100 cases of GC and paired adjacent non-cancerous tissue was purchased from Shanghai Outdo Biotech (HStmA180Su08). For IHC, the target molecule was performed on tissue microarray chips using IKKε antibody (Invitrogen,# PA5-15439). The microarray were stained with immunohistochemical streptavidin-peroxidase (SP) staining. The immunostaining index was based on the proportion of positively stained tumor cells and staining intensity. The proportion of positively stained tumor cells was graded as (no positively stained cells), 1 (<10%), 2 (10%–50%), and 3 (>50% of positive cells), and staining intensity was scored as 0 (no staining), 1 (light yellow), 2 (yellow brown), and 3 (brownish-yellow staining). The immunostaining index was then calculated as the staining intensity score multiplied by the proportion of positively stained tumor cells; tumors with indexes of 0 to 2 were considered immunostaining-low and those with 3 to 9 were scored immunostaining-high. Tumor stage was reassessed according to the seventh edition of the UICC/AJCC TNM staging system.

### RNA interference analysis

IKKε shRNAs (Shanghai Genechem Co., Ltd) were used to knock down IKKε according to the protocols provided by the manufacturer.

### Western blotting

Immunoblots were performed following the previously described procedures. The following antibodies were used in the study [22]: IKKε (Cell Signaling Technology, #3416) and β-actin (Cell Signaling Technology, #4970).

### Cell migration and invasion assays

*In vitro* invasion and migration assays were performed in 24-well Boyden chambers (Corning Incorporated, Corning, NY, USA) with or without Matrigel (BD) pre-coating. First, 600 ml of complete medium was added to the lower chamber. Second, 200 ml of a 2×10^5^/ml cell suspension prepared in FBS-free Dulbecco’s Modified Eagle’s Medium (DMEM) was seeded into the top well of the insert, and the cell migration filter was inserted into the lower chamber; the wells were incubated for 12 to 48 h at 37°C. Then, the cells on the top side of the filter were removed, the invasive and migrating cells were fixed with 500 ml of 4% paraformaldehyde for 20 min, and the fixed cells were stained with hematoxylin for 3 min. The invasive and migrating cells were counted and photographed under a light microscope. All experiments were conducted in triplicate.

### Cell proliferation

Cell proliferation assay was performed using Cell Counting Kit -8 reagent (Whsbio, Beijing, China) according to the manufacturer’s instructions.

### Clone formation assay

A total of 200 cells were seeded onto wells of a 6-well culture plate. The cells were then incubated for 12 days and subsequently stained with Giemsa solution. The number of colonies containing ≥50 cells in the plates was determined using the formula: plate clone formation efficiency = (number of colonies/number of cells inoculated) × 100%.

### Wound closure assay

Six-well plates were used to seed GC cells at a density of 2 ×10^5^ cells per well. Then, cells were scratched with a sterile pipette tip upon confluence. An inverted microscope was used to observe wound closure at 0 h and 24 h.

### Assessment of melanoma lung metastasis

After confluence of culture in ordinary DMEM, B16-F10 were delivered to IKKε null mice and wild-type (WT) mice (purchased from The Jackson Laboratory) by tail-vein injection (2×10^5^ cells/mouse). Lung melanoma metastases were quantified by counting the number of colonies. T-cells in bronchoalveolar lavage fluid (BALF) were analyzed by flow cytometry with antibodies against CD8.

### Xenograft model

Subcutaneous xenografts were created in the flank regions of 4-week-old nude male mice (8 mice per cell line). A total of 5 ×10^6^ MGC-803 cells were implanted with or without IKKε alteration by shRNA transduction. Monitoring of tumor nodules was performed every 4 days, and tumor volumes were estimated with the following formula:Volume = width × length × (width + length)/2. The mice were euthanized on day 28,and tumors were removed. The animal studies were approved by the Nanjing Medical University Ethics Review Board.

### The metastasis of GC cells *in vivo*

Stable SGC-7901 cells (transfected with IKKε shRNA or Control) were injected into nude mice via the tail vein, and the mice were sacrificed 6 weeks later for the analysis of the invasive lesions in the lungs.

### Statistical analyses

The data are presented as the means ± standard deviation (SD). Independent Student *t*-tests were used to compare the continuous variables between the 2 groups, and categorical variables were compared using the Chi-squared test. Overall survival was calculated using the Kaplan-Meier method and the log-rank test. Multivariate analysis was performed using the Cox proportional hazards model. Differences were considered significant if *P*<0.05.
